# The complete chloroplast genome of *Euphorbia prostrata* (Euphorbiaceae)

**DOI:** 10.1080/23802359.2024.2313039

**Published:** 2024-02-08

**Authors:** Ami Oh, Eun Su Kang, Dong-Chan Son, Soo-Rang Lee

**Affiliations:** a Department of Biology Education, Chosun University, Gwangju, Republic of Korea; b Division of Forest Biodiversity, Korea National Arboretum, Pocheon, Republic of Korea

**Keywords:** *Euphorbia*, *euphorbia prostrata*, Euphorbiaceae, phylogenetic analysis, plastome

## Abstract

*Euphorbia prostrata* Aiton 1789, an annual herb, is native to tropical and subtropical Americas and was introduced into many parts of the Old-World including Korea. We determined the genomic characteristics of the complete chloroplast genome of *E. prostrata* with a *de novo* assembly strategy. The complete chloroplast genome was 162,858 bp long, and harbored 86 protein coding, 37 tRNA and 8 rRNA. The genome showed the typical quadripartite structure consisting of a large single copy (LSC) (90,580 bp), a small single copy (SSC) (18,570 bp) and a pair of inverted repeats (IRs) (26,854 bp). The phylogenetic tree inferred from chloroplast genomes of 25 taxa, which belong to Euphorbiaceae indicated that the *Euphorbia* is a monophyletic group and *E. prostrata* is closely related with *E. humifusa*, *E. thymifolia* and *E. hirta,* forming a well-supported clade. Our findings can be valuable for understanding the phylogenetic relationships and the evolution in Euphorbiaceae and will provide basic information for future studies on *E. prostrata* from genomic perspective.

## Introduction

**Figure 1.**
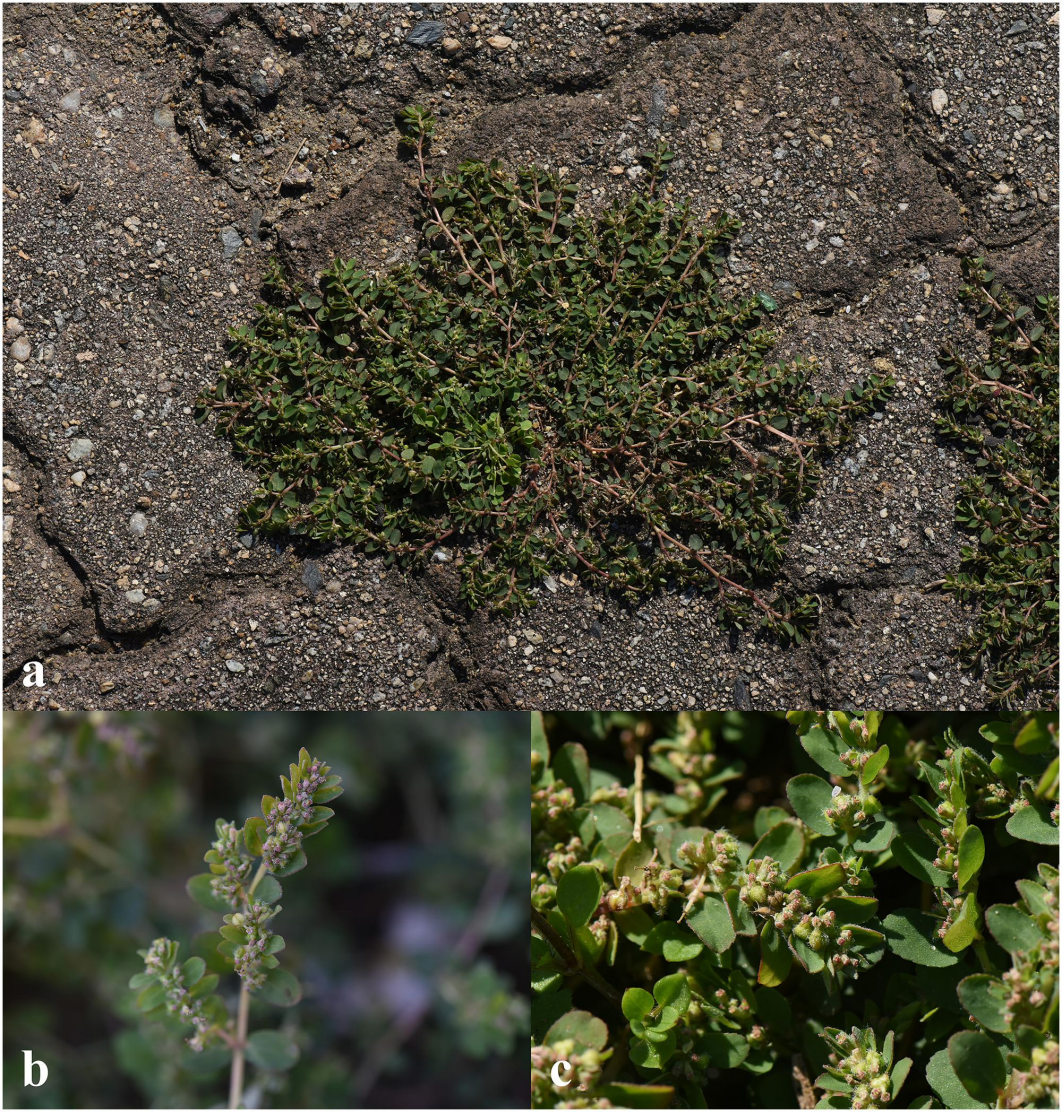
Photographs of *Euphorbia prostrata* Aiton. (a) whole plant, (b) flowers and (c) fruits. Stems many and prostrate; leaves opposite; cyathia with many male flowers and a single female flower; fruits 3-lobed. Photographs were taken by Eun Su Kang in Mokpo-si, South Korea.

*Euphorbia prostrata* Aiton 1789 (Euphorbiaceae) is an annual herb which has a prostrate stem and grows on sandy, trampled ruderal areas, along railroad tracks, roads or between rocks in gardens and fields (Yang et al. 2008; Milan et al. 2016) (Figure 1). *Euphorbia* (∼2,000 species) is one of the largest genera comprising four subgenera, *Rhizanthium*, *Esula*, *Euphorbia* and *Chamaesyce* (Horn et al. 2012). Subgenus *Chamaesyce* that *E. prostrata* belongs to is the second-most species-rich group and is mainly distributed in New World encompassing about 600 species worldwide (Yang et al. 2012). The species is native to tropical and subtropical region of Americas and was introduced into many parts of Old World including Korea (Ma et al. 2008). Although the plant was introduced and posed threat to natural ecosystems in many Eurasian countries, the genomic information applicable to eco- and evolutionary studies that offer critical information for management plans is limited. In this study, we investigated the genomic architecture of the chloroplast genome of *E. prostrata* using whole genome shotgun sequencing technique to provide genomic information that can be used in related research and management strategy of the species.

## Materials and methods

Fresh leaves of *E. prostrata* were collected from Mokpo-si, South Korea (N 34°47′29.94.″, E 126°23′10.349.″) and stored with silica gel in a plastic Ziplock bag until further use. We prepared the voucher specimen and stored it at the Herbarium of Korea National Arboretum (KH), Pocheon, Korea (http://www.nature.go.kr, Hee Young Gil, E-mail: warmishe@korea.kr, Voucher number: AP210906). We isolated the genomic DNA from the silica dried leaf tissue with DNeasy Plant Mini Kit (Qiagen, Hilden, Germany) according to the protocol of manufactures. The quality of DNA was checked on a 1% agarose gel through gel electrophoresis running. Library preparation and the genome sequencing were conducted by Macrogen Co. (Seoul, Korea). The genome sequencing was performed on an Illumina Miseq platform (Illumina, San Diego, CA, USA) producing ten million high-quality 300 bp paired-end reads. After trimming bad quality reads, we performed *de novo* assembly using Geneious Prime v. 2022.1.1 (Kearse et al. 2012) based on the filtered 6,849,836 reads. Using GeSeq v. 2.03 (Tillich et al. 2017), we predicted the genes and manually curated it based on Blast search results. We identified the simple sequence repeats (SSR) with MISA (Beier et al. 2017). The search parameters for MISA analysis were set at ten repeat units for mono-nucleotides, six repeat units for di-nucleotides, and five repeat units for tri-nucleotides, tetra-nucleotides, penta-nucleotides, and hexa-nucleotides. The complete chloroplast genome of *E. prostrata* was deposited into the NCBI GenBank (accession no. ON631059).

To infer the phylogenetic relationship between *E. prostrata* and the other *Euphorbia* taxa, we aligned 23 chloroplast genomes of *Euphorbia* and two outgroup taxa in MAFFT v. 7.450 (Katoh et al. 2019). All sequences except for *E. prostrata* were downloaded from NCBI Genbank. We designated *Croton laevigatus* and *Croton tiglium* as outgroups based on a previous study (Wurdack et al. 2005). A Maximum likelihood (ML) phylogenetic tree was reconstructed using RAxML v. 4.0 with GTR GAMMA model. We used 1000 bootstrap replicates for the clade support.

## Results

**Figure 2.**
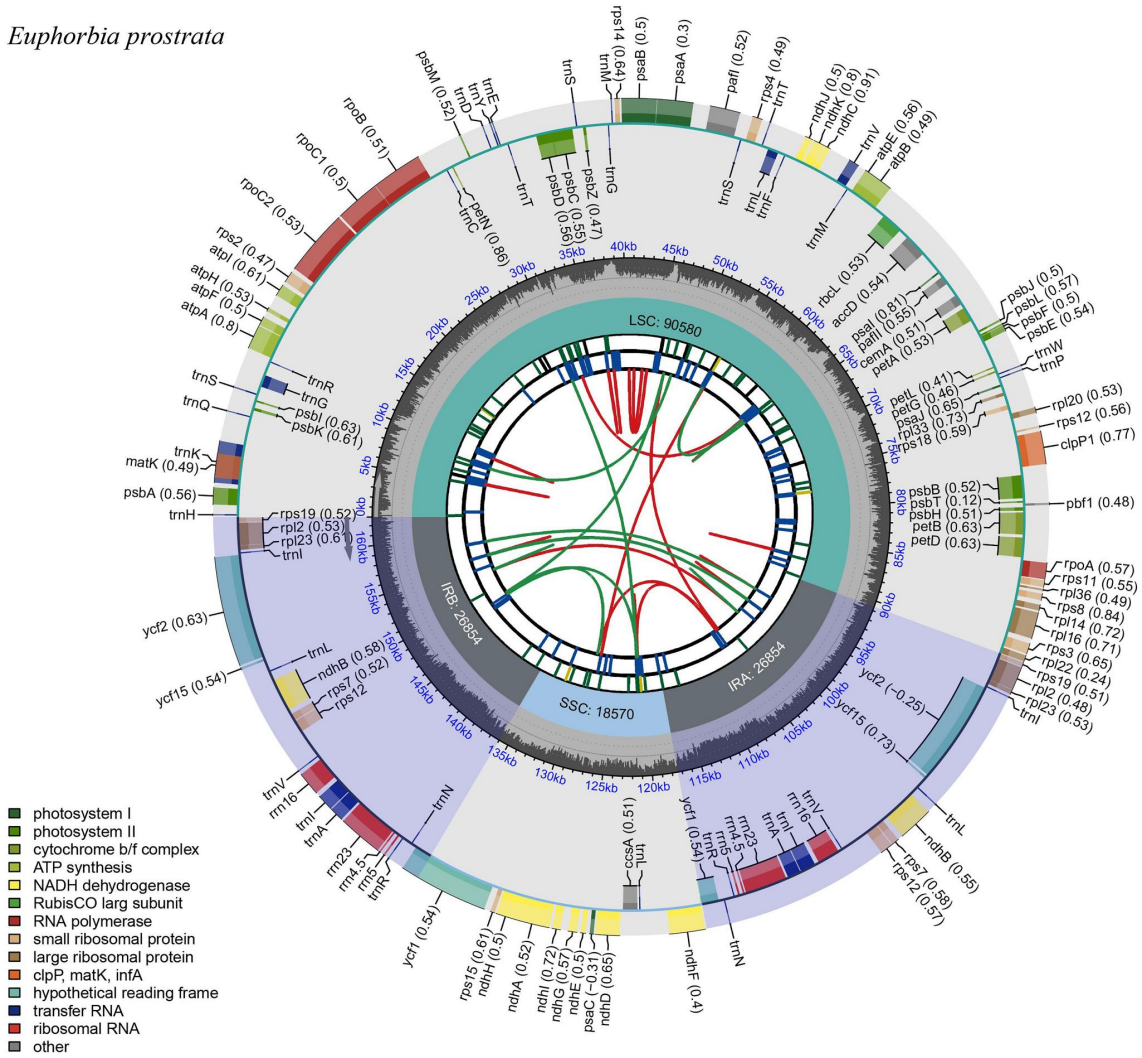
Circular map of *E. prostrata*. The map of complete chloroplast genome was generated using CPGView (http://www.1kmpg.cn/cpgview). The map consists of six tracks. From the center to the outer, the first track shows dispersed repeats connected by red and green arcs indicating the direction (forward and reverse, respectively). The second track shows long tandem repeats as blue bands, and the third track shows short tandem repeats or microsatellites as green bands. The fourth track represents the GC content along the plastome. Finally, the sixth track represents the genes as colored boxes, the inner boxes present clockwise transcription, and the outer boxes present counterclockwise transcribed genes. The optional codon usage bias is displayed in the parenthesis after the gene.

The complete chloroplast genome of *Euphorbia prostrata* was identified as 440,674 reads (4.27% of total raw data), and the average depth was 1573.32 × (Supplementary Figure 1). The complete chloroplast genome was 162,858 bp long with a GC content of 35.3%, and showed a typical quadripartite structure consisting of a large single copy (LSC: 90,580 bp), a small single copy (SSC: 18,570 bp) and a pair of inverted repeats (IR: 26,854 bp) (Figure 2). Overall, there were 131 genes containing 86 protein coding genes, 37 transfer RNA (tRNA) genes and 8 ribosomal RNA (rRNA) genes (Figure 2). The average GC content for the 131 genes was 38.8%. For the rRNAs and tRNAs, the GC contents were 55.4 and 52.9%, respectively, showing notably higher levels than the ones found in the other genes. The LSC region contained 60 coding sequences (CDS), 82 genes, and 22 tRNA, while the SSC region contained 12 CDS, 13 genes, and one tRNA. In IR, nine CDS, 20 genes, four rRNA, and seven tRNA were observed. The *rps12* is a trans-splicing gene (Supplementary Figure 2). Eleven genes including *rpoC1*, *pafl*, *clpP1*, *petB*, *petD*, *rpl16*, *rpl2*, *ndhB*, and *ndhA* are cis-splicing genes; *ndhB* and *rpl2* had two copies (Supplementary Figure 3).

We found 80 SSRs in chloroplast genome, most of which were mono-nucleotide repeats: 69 SSRs were mono-nucleotides, and 11 SSRs were di-nucleotides. In mono-nucleotide repeats, 35 repeats with T unit and 34 repeats with A unit were observed.

**Figure 3.**
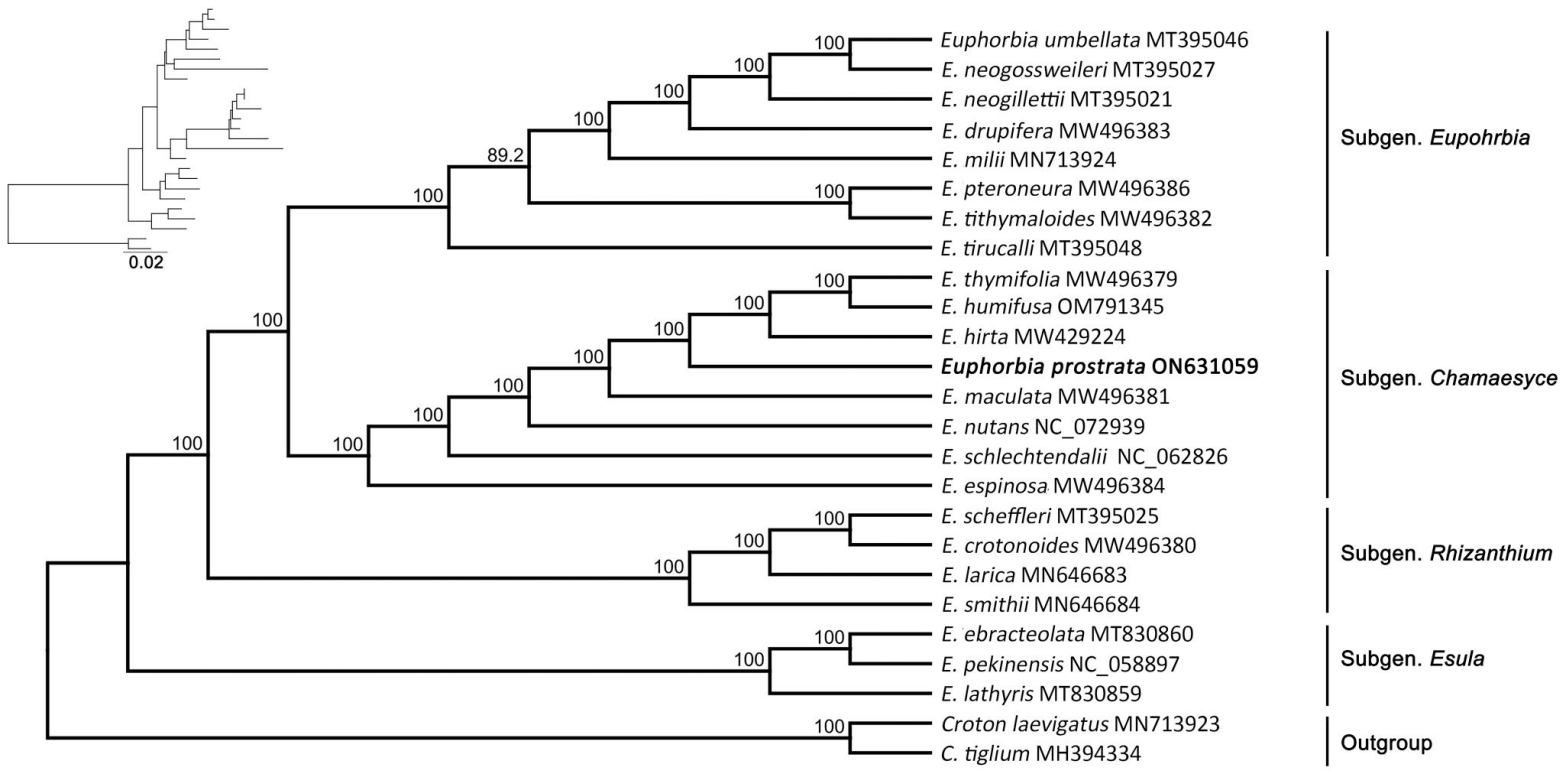
Maximum likelihood (ML) tree based on chloroplast genome sequences of 25 species from Euphorbiaceae, with numbers on the nodes indicating the bootstrap value (>50%). the sequences incorporated in the tree are as follows: *E. prostrata* ON631059 (this study), *E. umbellata* MT395046, *E. neogossweileri* MT395027, *E. neogillettii* MT395021, *E. drupifera* MW496383, *E. milii* MN713924 (Jiang et al. 2020), *E. pteroneura* MW496386, *E. tithymaloides* MW496382, *E. tirucalli* MT395048 (Patil et al. 2023), *E. thymifolia* MW496379, *E. humifusa* OM791345, *E. hirta* MW429224, *E. maulcata* MW496381, *E. nutans* NC_072939, *E. schlechtendalii* NC_062826, *E. espinosa* MW496384, *E. scheffleri* MT395025, *E. crotonoides* MW496380, *E. larica* MN646683 (Patil et al. 2023), *E. smithii* MN646684 (Chen et al. 2021), *E. ebracteolata* MT830860 (Wang et al. 2021), *E. pekinensis* NC_058897 (Wang et al. 2022), *E. lathyris* MT830859 (Ma et al. 2020), *Croton laevigatus* MN713923 (Liao et al. 2020), *C. tiglium* MH394334.

The ML tree confirmed that *E. prostrata* belongs to the *Euphorbia* (Figure 3). In the ML tree, the 23 species of *Euphorbia* formed a monophyletic group with strong support (BP = 100). *Euphorbia prostrata* formed a monophyletic group with *E. humifusa*, *E. thymifolia* and *E. hirta* (BP = 100), indicating that *E. prostrata* is closely related with these species. *Euphorbia prostrata* was closely related to the clade comprising *E. humifusa*, *E. thymifolia* and *E. hirta*. It is previously known that these four species all belong to the same subgenus *Chamaesyce*. In our phylogenetic tree, *E. prostrata* was not among the *Euphorbia* species that diverged early from the common ancestor determined herein.

## Discussion

The complete plastome data we assembled along with the previously reported data showed the resolution power of plastid genome data for phylogenetic studies of *Euphorbia* taxa (Webster 1994; Berry et al. 2005; Horn et al. 2012). *Euphorbia* species formed a monophyletic group diverging from the outgroups, and is subdivided into four distinct groups (Subgenera *Esula*, *Rhizanthium*, *Chamaesyce* and *Euphorbia*). Our ML analysis results are considered to effectively reflect their relationships and consistent with the results of Iwata et al. (2022). *Euphorbia prostrata* formed a clade with taxa from the subgenus *Chamaesyce*.

*Euphorbia* is a large-scale genus containing about 2,000 species (Horn et al. 2012), and well-known for its remarkable structural variability (Horn et al. 2012). The analysis of the chloroplast genome can be helpful in understanding their relationships and studying the phylogeny. The supplementary research that includes additional species is needed to achieve more significant results. Nevertheless, we are convinced that our findings proved that the plastome sequence data can offer basic information of plastome evolution and help inferring phylogenetic relationships and the species identification of Euphorbiaceae.

## Supplementary Material

Supplemental Material

Supplemental Material

Supplemental Material

## Data Availability

The genome sequence data that support the findings of this study are openly available in GenBank of NCBI at (https://www.ncbi.nlm.nih.gov/) under the accession no. ON631059. The associated BioProject, SRA and Bio-Sample numbers are PRJNA917811, SRR23086253 and SAMN32549699, respectively.
