# Supplementary figures and images for: The complete chloroplast genome of Euphorbia prostrata (Euphorbiaceae)
Source: Mitochondrial DNA B Resour. 2024 Feb 8;9(2):267–71. doi: 10.1080/23802359.2024.2313039 (PMC10860408; doi:10.1080/23802359.2024.2313039)

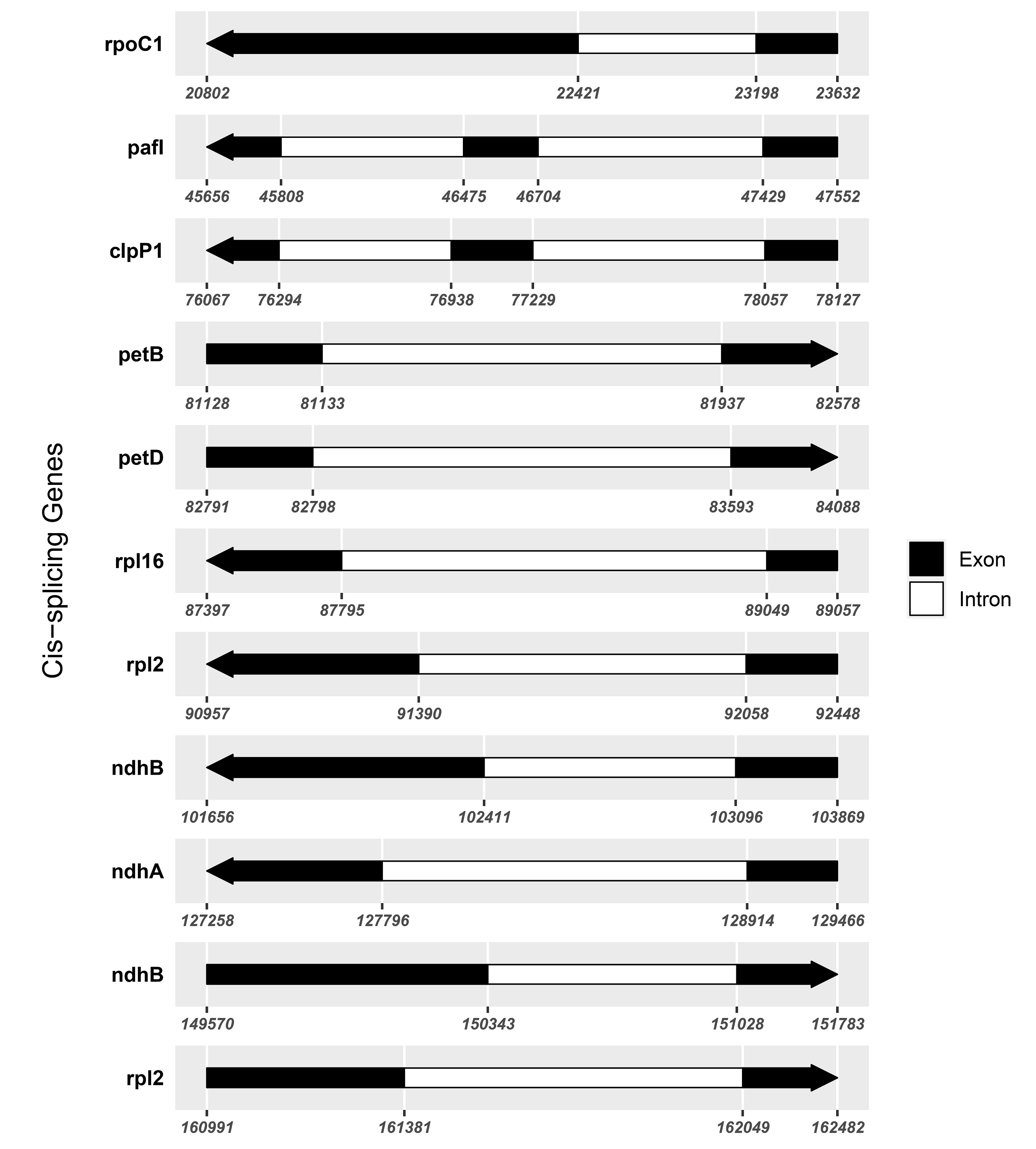

Supplement: Supplemental Material [file TMDN_A_2313039_SM3963.jpg]

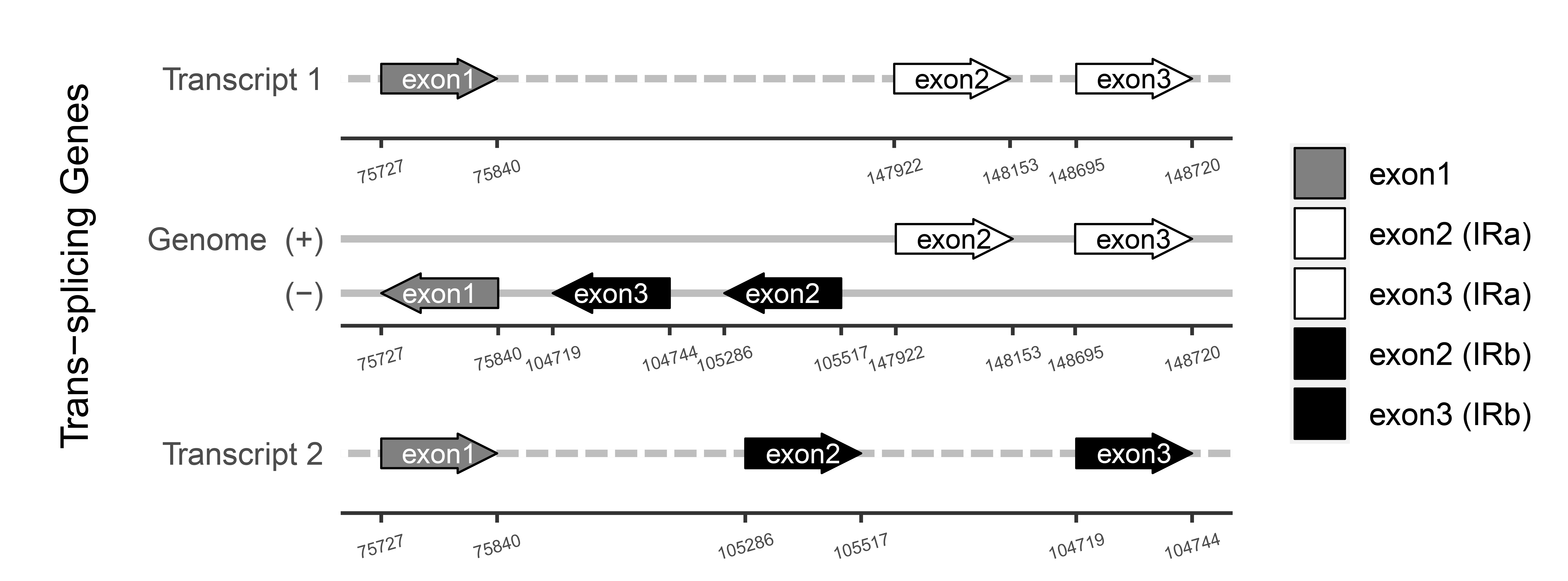

Supplement: Supplemental Material [file TMDN_A_2313039_SM3958.jpg]

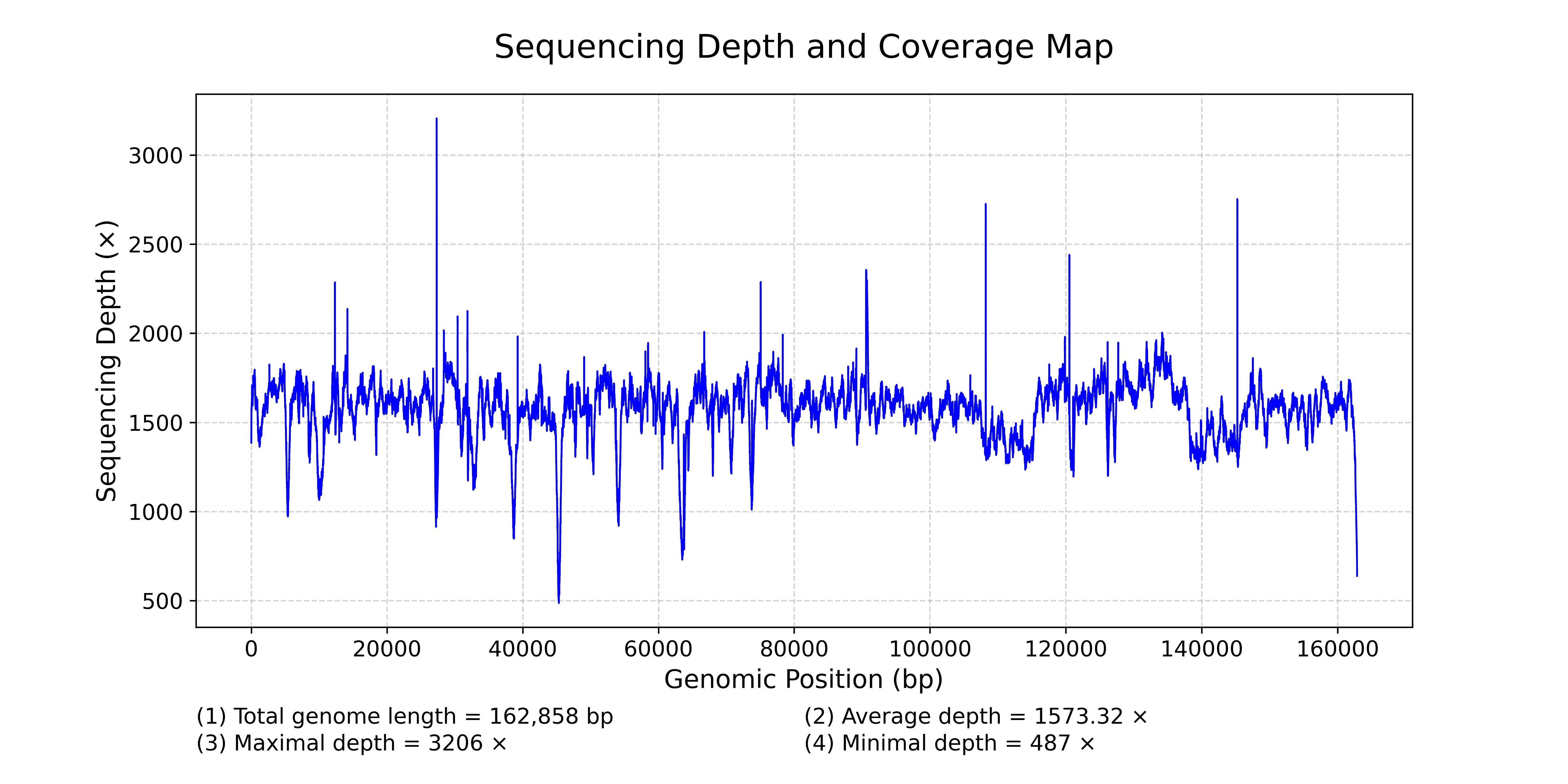

Supplement: Supplemental Material [file TMDN_A_2313039_SM3957.jpg]
